# PHENOL VERSUS LIDOCAINE IN OBTURATOR NERVE NEUROLYSIS FOR HIP JOINT PAIN

**DOI:** 10.1590/1413-785220233103e266865

**Published:** 2023-09-08

**Authors:** Chiara Maria Tha Crema, Luiza Previato Trevisan Magario, Wilian Carlos Siena, Nicole Marques Favato, Thabata Pasquini Soeira, Marcelo Riberto

**Affiliations:** 1 Hospital das Clínicas, Faculdade de medicina da USP de Ribeirão Preto, SP, Brazil.

**Keywords:** Phenol, Lidocaine, Osteoarthritis, Hip, Chronic Pain, Nerve Block, Fenol, Lidocaína, Osteoartrite do Quadril, Dor Crônica, Bloqueio Nervoso

## Abstract

**Introduction:**

For patients with severe hip osteoarthritis without clinical or socioeconomic conditions for total hip replacement, the obturator nerve block may serve for pain control and functional improvement. Either lidocaine or phenol are used, although the latter is expected to last longer. Objectives: Compare hip pain and functional performance after obturator nerve block with phenol versus lidocaine in patients with severe hip osteoarthritis who failed conservative treatment.

**Methodology:**

Forty-four patients scheduled for total arthroplasty due to severe osteoarthritis were randomized to the anterior branch of the obturator nerve with phenol (PG) or 1% lidocaine (LG), guided by electrical stimulation. Patients were evaluated with VAS, WOMAC, and pressure pain dolorimetry before the procedure and in the first and fourth months afterward.

**Results:**

Both groups improved significantly in pain control, pressure dolorimetry and functioning in the first month with reduced effect after 4 months, although the scores were still better than baseline. No statistical difference could be noticed between the groups. Severe adverse effects were not reported.

**Conclusion:**

Both lidocaine and phenol are equally effective and safe in the obturator nerve block for the control of pain and improvement in functioning in patients with severe hip OA. ***Evidence Level I; Randomized control trial, double-blind*** .

## RESUMO

**Introdução:**

Em pacientes com osteoartrite grave do quadril, sem condições clínicas ou socioeconômicas para a substituição total do quadril, o bloqueio do nervo obturador pode servir para o controle da dor e ganho funcional. Pode-se usar lidocaína ou fenol, embora seja esperado que o último apresente maior duração.

**Objetivo:**

Comparar a dor no quadril e o desempenho funcional após o bloqueio do nervo obturador com fenol versus lidocaína em pacientes com osteoartrite grave do quadril que não obtiveram sucesso no tratamento conservador.

**Metodologia:**

Quarenta e quatro pacientes programados para artroplastia total devido à osteoartrite grave foram randomizados para o ramo anterior do nervo obturador com fenol (PG) ou lidocaína a 1% (LG), guiados por estimulação elétrica. Os pacientes foram avaliados com EVA, WOMAC e dolorimetria de dor por pressão antes do procedimento e no primeiro e quarto meses seguintes.

**Resultados:**

Ambos os grupos apresentaram melhora significativa no controle da dor, na dolorimetria por pressão e na funcionalidade no primeiro mês, com efeito reduzido após quatro meses, embora as pontuações ainda fossem melhores do que a linha de base. Não foi possível observar nenhuma diferença estatística entre os grupos. Não foram relatados efeitos adversos graves.

**Conclusão:**

Tanto a lidocaína quanto o fenol são igualmente eficazes e seguros no bloqueio do nervo obturador para o controle da dor e melhora da funcionalidade em pacientes com OA grave de quadril. ***Nível de evidência I; Estudo clínico randomizado,duplo cego*** .

## INTRODUCTION

The main symptom of osteoarthritis (OA) is joint pain, tipically worsened by movement or load, but also present at rest, and accompanied by joint stiffness that lasts less than thirty minutes or joint instability, limitation of the range of motion, and physical disability. These may lead to a compromised functional capacity of the affected individual and give rise to changes in gait and activities of daily living (ADLs).^1^

Comprehensive rehabilitation therapy aims to control pain, improve mobility, and bring functional restoration. Therapeutic resources may include non-pharmacological strategies such as exercise, modalities, walking aids, and drugs such as analgesics, anti-inflammatories, opiates, capsaicin cream, injections with glucocorticoids or hyaluronic acid.^1^ Despite not being present in the therapeutic guidelines for this clinical condition, nerve blocks are a valuable interventionist resource, particularly when clinical treatment fails and the surgical indication for total hip arthroplasty is restricted due to the clinical conditions related to high surgical risk in the elderly patient with multiple comorbidities,2 or socioeconomic conditions.3 In this context, the obturator nerve block can be an analgesic therapeutic alternative that enables the rehabilitation process.^4 – 6^

Nerve blocks interrupt the nociceptive input at its origin, blocking conduction by the spinal, cranial nerves, or afferent fibers that accompany the autonomic nerves. It is an indication for the relief of multiple painful syndromes, of nociceptive or neuropathic nature.^7^ Among the substances used in the practice of these blocks are lidocaine and phenol, which share the immediate local anesthetic action, which is more prolonged in the later due to their immediate selective effect on smaller nerve fibers, resulting from the destruction of small vessels, which initially saves large fibers.^8^ John Monagle and Joanne Ee described the use of intra-articular phenol in hip osteoarthritis, where they achieved better pain control and improved functioning^6^ and a previous study by our group, carried out only with the use of phenol in a series of patients,4 there was an improvement in pain, especially during the first two months after the block.^9^

This study aims to evaluate the effectiveness of a pain treatment done by applying phenol to the anterior branch of the obturator nerve in comparison with the application of lidocaine in patients with hip osteoarthritis, who did not improve with the conservative treatment.

## METHODS

This study was approved by the institution Internal Review Board (CAAE: 66553517.8.0000.5440), all subjects were instructed on the risks and benefits and signed an informed consent form prior to the start of the study.

**Figure 1:**
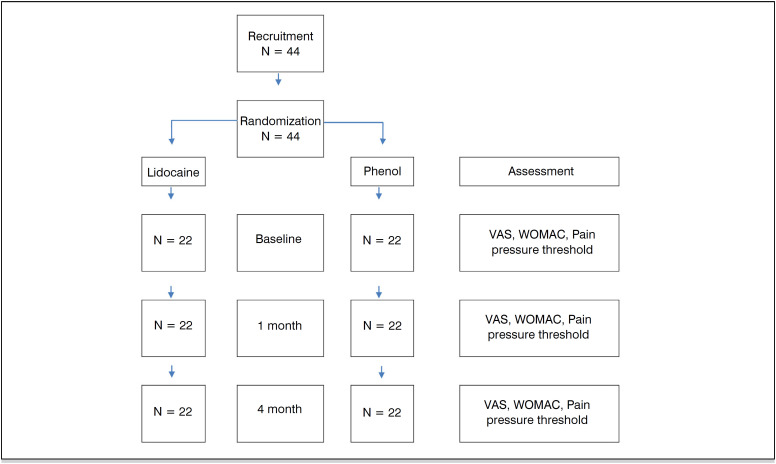
Flowchart of allocation of subjects.

This was a randomized, double-blind clinical trial. Participants were recruited from the rehabilitation center of a tertiary general hospital from Brazil's public health system. Inclusion criteria for this study were: 1) both sexes, 2) adults, 3) diagnosis of severe hip OA, based on the stage of joint degeneration(Kellgren Lawrence class 3 or 4), 4)failed conservative treatment such as drugs, physical therapy exercises, injections with glucocorticoids or hyaluronic acid, pain intensity assessed by the Visual Analog Scale (VAS) greater than six, 5) no known phenol allergy or uncontrolled coagulopathy. Exclusion criteria consisted of the presence of generalized pain, inability to undergo the block procedure under electrical stimulation guidance, due to pacemakers or other implanted devices sensitive to electrical currents, and difficulty in understanding the assessment instruments. Figure 1 shows the flowchart of allocation of individuals.

Forty-four severe hip OA patients were randomized in blocks of four participants with a computer-generated list (website www.randomization.com)for the blockade in the anterior branch of the obturator nerve (BABON)either with phenol (group PG) or lidocaine (group LG).

In this study, patients were evaluated immediately after randomization and before the nerve block, follow up assessments were done one and four months after the intervention. Assessment used pain intensity VAS, which consisted of a 100 mm straight line anchored at the extremities to the expressions ‘no pain’ and ‘worst possible pain’ on which the patient is asked to indicate the intensity of the painful symptom during the day of evaluation. Dolorimetry consisted in the use of a pressure dynamometer with a cylindrical and rubberized tip of 1 cm2 to inflict progressive pressure on myofascial trigger points until the patient manifested pain^10^ – the painful pressure threshold indicate the sensibilization of that specific trigger point, thus lower scores indicated more sensitive points which needed less pressure to cause pain. The questionnaire Western Ontario and McMaster Universities Arthritis Index (WOMAC) was used to assess pain, stiffness, and physical function specifically for hip conditions, having already been used in several RCTs for drug and surgical treatment of hip OA.^11^

**Figure 2:**
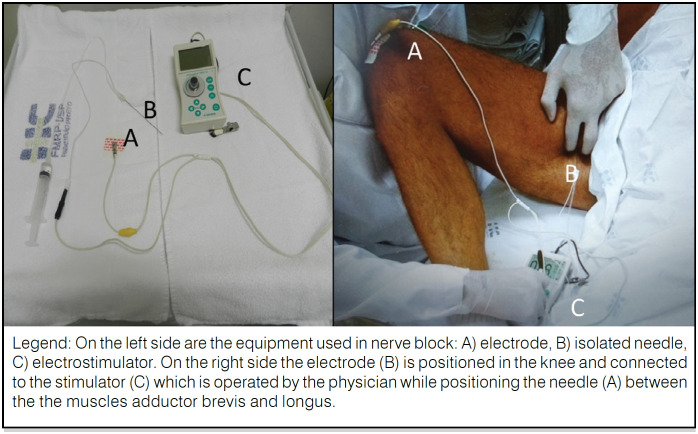
Equipment and positioning of lower leg for blocking of the anterior branch of the obturator nerve.

Using manual palpation, the interval between muscles adductor longus and brevis was identified, and needles were inserted 3 to 5 centimeters distal to their upper extremity. The anterior branch of the obturator nerve could be localized with 100mm-long isolated needles connected to an electrostimulator.^12^ Figure 2 shows the arrangement of this localization system: the electrical current produced by the stimulator would travel from an electrode to the tip of the needle. Electrical current as low as 2 mA can produce muscle contraction. When the best contraction of adductor muscles was obtained with 1 mA, which is the rheobase for peripheral nerves, successful localization was accomplished. Treatment was performed with an application of 2.5 ml of phenol 6% or lidocaine 1% to the anterior branch of the obturator nerve according to a randomization list. Immediate effect is the interruption of muscle contraction.

Both lidocaine and phenol solutions are transparent liquids, but the later exhalates a pungent smell and could be easily differentiated from the first. To warrant blinding, the therapeutic solutions were prepared by a research nurse who was the only one in contact with the randomization sequence. She would bring the syringes with an open bottle of phenol, thus the injection would always be performed in a phenol smelling environment.

The sample size calculation was based on the result of a previous study published by Crema et al., in which a series of patients with severe hip OA underwent neurolysis in the anterior branch of the obturator nerve to control pain, having the mean pain intensity (VAS) varied from 8.2 ± 0.9 at baseline to 6.6 ± 1.7 at the end of one month, 6.5 ± 1.7 at the end of two months, and 7.3 ± 1 at six months (p= 0.0094). Considering an effect size of 10%, the statistical power of 80% and the significance level of 0.05, twenty participants would be needed in each study group, to which a margin of 10% was added (four more participants) for the case of follow-up losses. Quantitative variables were evaluated with mean and standard deviation, whereas in categorical variables, percentages were evaluated. After verifying the normality of the distribution of variables, the evaluation of the results of pain assessment in patients with the VAS (primary outcome), WOMAC and its subscales and dolorimetry, the ANOVA test for repeated measures was used to assess the evolution of the values of these variables. As the dolorimetry was always evaluated in a group of six muscles, adductor magnus, short and long, gluteus minimus, medius and piriformis, we preferred to create an index of mean value of these points rather than study them individually. The analysis of the results was based on the intention to treat.

## RESULTS

Forty-four patients were included in the study according to the flowchart shown in Figure 1 , 22 (50%) of whom were men. The mean age of the entire sample is 54.6 ± 15.7 years. Table 1 presents the biodemographic and clinical data.

**Table 1:** Biodemographic and clinical data.

	All	Phenol	Lidocaine
N	44	22	22
Men (%)	22	11	11
Age (Years)	54.6±15.7	55.9±16.8	53.2±14.7
**RX Classification**			
Class 3	21 (47.7%)	11 (50.0%)	10 (55.5%)
Class 4	23 (52.3%)	11(50.0%)	12 (54.5%)

Idiopathic hip OA was responsible for 50%, followed by avascular necrosis of the femoral head (22.7%). Other etiologies of hip disease were Legg-Perthes and rheumatoid arthritis.

**Figure 3:**
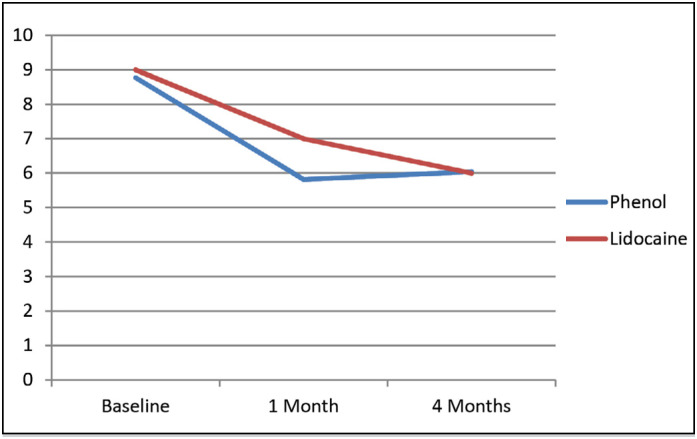
Evolution of pain intensity assessed by VAS during the study in patients blocked with phenol and lidocaine.

Figure 3 shows pain intensity reported by VAS during the study. Baseline pain intensity was similar in both groups (phenol: 87.0 ± 15.0 x lidocaine90.0 ± 11.0; p>0.05). After one month of a single nerve block, pain intensity reduced in both groups, although slightly more in those subjects injected with phenol, without statistical difference (phenol: 58.0 ± 29.0 x lidocaine: 70.0 ± 27.0; p>0.05), and both groups finished the follow-up period with very similar pain intensities (phenol: 59.0 ± 29.0 x lidocaine: 60.0 ± 32.0; p>0.05). A significant decrease in pain levels over the course of follow-up was demonstrated by ANOVA.

**Figure 4:**
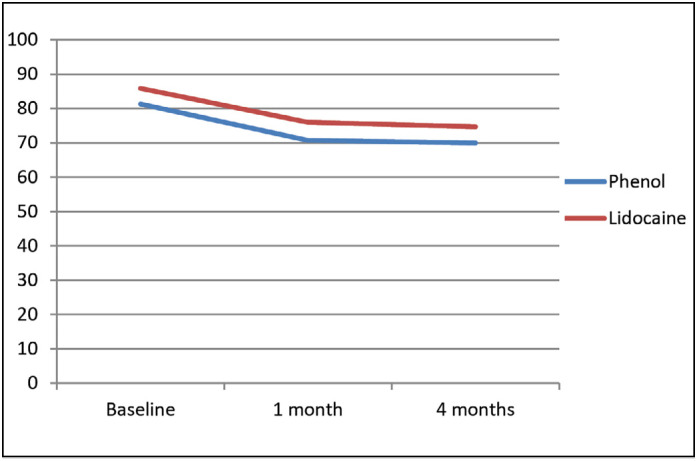
Evolution of functioning assessed by WOMAC during the study in patients blocked with phenol and lidocaine.

Similar results concerning functioning can be observed in Figure 4 . Again, both groups had similar baseline scores and decreased the compromise in quality of life after one month and four months, without statistical difference.

Table 2 shows the evolution of the mean values of each domain of the WOMAC questionnaire. For the pain component, no differences were identified between the groups. For the group with phenol, there was pain reduction at the end of one month, but with resumption of pain levels in the fourth month; on the other hand, in the lidocaine group, the reduction in the pain component values of this questionnaire obtained at the end of the first month was maintained until the end of the observation period. The ANOVA test to verify the interaction of the type of treatment with the temporal evolution of this component was not significant. For the ‘stiffness’ and ‘function’ components, both groups had improved indices at the end of the first month of the segment, with stability of gains at the end of four months for both variables in the group in which phenol was used, while for ‘stiffness’ there was a progressive improvement in the lidocaine group, but this was not the case for the ‘function’ component. Again, the ANOVA test did not identify a statistically significant interaction between treatment and evolution over the observation period for these two questionnaire components.

**Table 2:** Detailed evolution of WOMAC components during the study in patients blocked with phenol and lidocaine.

	Treatment	Baseline	One month	Four months
WOMAC		83.6 ± 18.3	81.3 ± 12.9	85.9 ± 5.5
Pain		17.4 ± 2.7	16.8 ± 3.1	18.0 ± 2.0
	Phenol	16.8 ± 3.1	13.9 ± 3.6 *	16.5 ± 12.5
	Lidocaine	18.0 ± 2.0	15.4 ± 3.0 *	15.1 ± 2.9 *
Stiffness		5.8 ± 1.8	5.6 ± 2.1	6.1 ± 1.6
	Phenol	5.6 ± 2.1	4.2 ± 2.3 *	3.2 ± 2.7 *
	Lidocaine	6.1 ± 1.6	4.4 ± 2.2 *	3.0 ± 2.5 * §
Function		60.6 ± 6.8	59.1 ± 8.2	62.2 ± 4.7
	Phenol	59.1 ± 8.2	52.3 ± 11.5 *	52.7 ± 10.4 *
	Lidocaine	62.2 ± 4.7	56.5 ± 7.7 *	57.1 ± 5.2 *

* Legend: p<0.05 in relation to the initial value, and

§ p<0.05 in relation to the value one month after the beginning of the treatment.

**Figure 5:**
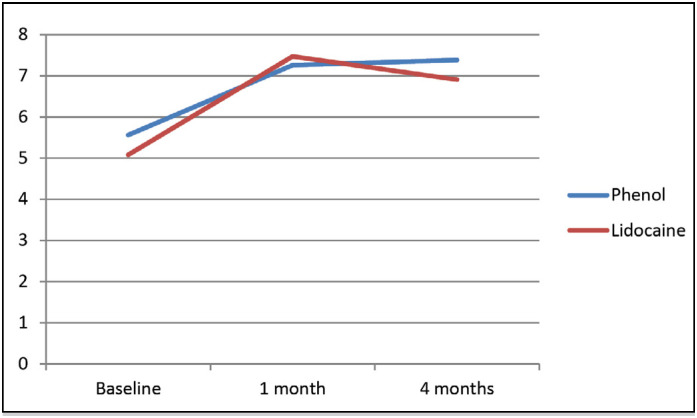
Dolorimetry evolution.

Mean dolorimetry values were calculated from the pain pressure theshold obtained in the medial gluteus medius, lateral gluteus medius, gluteus minimus, and piriformis. Although the curves in Figure 5 are inverted in comparison to figures 3 and 4, the meaning is the same, baseline pain pressure thresholds were similar and improved after one and four months, but without statistical difference among the groups.

Patients did not report adverse effects after the procedure, such as paresthesia, bruising or pain.

## DISCUSSION

This randomized controlled trial was successful in comparing the effect of blocking the anterior branch of the obturator nerve with lidocaine and phenol in patients with severe hip OA. Overall, it was possible to demonstrate a reduction of about 33% in pain intensity assessed by the VAS for up to sixteen weeks in these patients who were candidates for surgical treatment, accompanied by an improvement in quality of life and functioning measured by WOMAC. However, there was no significant change in pressure dolorimetry. The two pharmacological agents produced very similar results in all parameters evaluated, with minimal differences. Alternatively, Silva et al. describe a case report in which they performed an obturator nerve block with 10 ml of 0.25% bupivacaine, resulting in 100% improvement of pain and improvement in the patient's functioning, who started presenting independence for daily activities. The analgesic effect persisted for 40 days.^14^

The initial hypothesis that the effects of phenolblock would last longer was not confirmed. Contrasting to lidocaine, which effect of neural block lasts 2 to 4 hours, the effects of phenolic blocks are based in the local anesthetic action on gama fibers, reducing the spastic reflex associated to pain. Also, this substance can produce axoniotmesis, which is the disorganization of the structure of myelin sheath of axons, without injury to endoneural tubes, which may reduce motor inputs and cause relaxation. Its effect in muscle relaxation and spasticity control is well known.^9^ The effects of chemical neurolysis with phenol are not permanent, since functional reinnervation may occur in a period of months. or years.^8^ The time of action of this procedure may vary with the concentration of phenol, injected volume, duration of exposure, and injection technique. In a study carried out by Felsenthal, the degree of conduction block differed with different concentrations and volumes of phenol injected up to eight weeks after the nerve block, which could explain the variation in duration.^15^

The WOMAC questionnaire showed that, in an unified way, up to the fourth month there was an improvement of joint stiffness, feeling of instability or joint insecurity, limited range of motion, and physical incapacity leading to impairment of activities such as walking, sitting, standing, and performing physical activities.

None of the individuals in this study developed sensory changes or neuropathic pain pattern in the sensory territory of this nerve branch - the medial face of the thigh, although it is expected that, when injected close to nerves with a predominance of sensory fibers, phenol may cause dysesthesia or anesthesia for up to four months, and eventually this sensation can be described as neuropathic pain, with terms such as shock and burning and with constant or paroxysmal presentation. The most frequent adverse effects in phenolysis are: dysesthesia and pain resulting from a local inflammatory process, ranging from 0.4% to 5% in children and 2-32% in adults.^13^

This study has some limitations, such as the evaluation of functionality through a questionnaire to be answered by the patient and not through physical tests; the patients had multiple comorbidities and presented arthritis in other joints as a confounding factor in the perception of improvement; and also the presence of periarticular pain pathologies should be investigated. Special attention should be directed to muscular affections, such as myofascial pain, as its treatment can represent a significant symptomatic relief.^16^ The most frequently involved muscles are the piriformis, iliopsoas, adductor longus, gluteus medius and minimus, adductors, and the piriformis muscle, which is related to pain over the buttock, along with its insertion in the greater trochanter and radiating to the posterior surface of the thigh. The iliopsoas muscle, in turn, presents a distribution of pain associated with its trigger points on the anterior and proximal surface of the thigh, as shown in an unpublished study by Magário et al.^17^

Given that there is a lack of studies on blocks aimed at improving pain in hip osteoarthritis, the positive aspects of this study include the assessment of methods based on blocks to relieve pain and improve the quality of life or functionality for patients with few resources.

## CONCLUSION

The application of phenol or lidocaine in the anterior branch of the obturator nerve can alleviate pain and improve the functionality of patients with hip OA, and may be an alternative treatment for patients who have not undergone THA surgery, either because they are not in clinical condition or because of the queue waiting for the procedure.
